# Stratifying the risk of ovarian cancer incidence by histologic subtypes in the Korean Epithelial Ovarian Cancer Study (Ko‐EVE)

**DOI:** 10.1002/cam4.5612

**Published:** 2023-02-15

**Authors:** Soseul Sung, Youjin Hong, Byoung‐Gie Kim, Ji‐Yeob Choi, Jae Weon Kim, Sang‐Yoon Park, Jae‐Hoon Kim, Yong‐man Kim, Jong‐Min Lee, Tae Jin Kim, Sue K. Park

**Affiliations:** ^1^ Department of Biomedical Sciences Seoul National University Graduate School Seoul Republic of Korea; ^2^ Department of Preventive Medicine Seoul National University College of Medicine Seoul Republic of Korea; ^3^ Cancer Research Institute Seoul National University Seoul Republic of Korea; ^4^ Integrated Major in Innovative Medical Science Seoul National University College of Medicine Seoul Republic of Korea; ^5^ Department of Obstetrics and Gynecology, Samsung Medical Center Sungkyunkwan University School of Medicine Seoul Republic of Korea; ^6^ Institute of Health Policy and Management Seoul National University Medical Research Center Seoul Republic of Korea; ^7^ Department of Obstetrics and Gynecology Seoul National University Hospital, Seoul National University Seoul Republic of Korea; ^8^ Center for Gynecologic Cancer National Cancer Center Goyang Republic of Korea; ^9^ Department of Obstetrics and Gynecology Gangnam Severance Hospital, Yonsei University College of Medicine Seoul Republic of Korea; ^10^ Department of Obstetrics and Gynecology, College of Medicine University of Ulsan, Asan Medical Center Ulsan Republic of Korea; ^11^ Department of Obstetrics and Gynecology Kyung Hee University Hospital at Gangdong Seoul Republic of Korea; ^12^ Department of Obstetrics and Gynecology Konkuk University School of Medicine Seoul Republic of Korea

**Keywords:** epidemiology, ovarian neoplasms, pathology, risk factors

## Abstract

**Introduction:**

This study aimed to verify the association between ovarian cancer (OC) and reproductive‐ and lifestyle‐related risk factors stratified by the subtype of OC.

**Methods:**

In this matched case–control study derived from the Korean epithelial ovarian cancer study (Ko‐EVE), we calculated the risk of OC subtypes using odds ratios (ORs) and 95% confidence intervals (95% CIs) in a logistic regression model.

**Results:**

As a result of matching, 531 cases and 2,124 controls were selected. Smoking had positive association with high‐grade serous (HGS) OC (OR = 2.69, 95% CI = 1.15–6.30), whereas alcohol consumption had positive association with mucinous type (MUC) (OR = 3.63, 95% CI = 1.39–9.49). Obesity (≥30 kg/m^2^) was associated with clear cell type (CLC) (OR = 4.57, 95% CI = 1.06–19.77). Spontaneous abortion was negatively associated with CLC (OR = 0.34, 95% CI = 0.13–0.90), in contrast to HGS (OR = 1.43, 95% CI = 0.96–2.15). Tubal ligation, hysterectomy, and oophorectomy were associated with decreased risk of HGS (OR = 0.14, 95% CI = 0.05–0.39; OR = 0.23, 95% CI = 0.07–0.73; OR = 0.28, 95% CI = 0.08–0.97, respectively). Early menarche was strongly associated with increased risk of CLC, but not MUC (OR = 6.11, 95% CI = 1.53–24.42; OR = 3.23, 95% CI = 0.98–10.86). Further, childbirth (≥2 times) was negatively associated with endometrioid type OC and CLC (OR = 0.11, 95% CI = 0.04–0.35; OR = 0.12, 95% CI = 0.02–0.37, respectively). Oral contraceptives and hormone replacement therapy were negatively associated with OC (OR = 0.61, 95% CI = 0.40–0.93; OR = 0.51, 95% CI = 0.32–0.80, respectively), and similar negative associations were also observed in HGS (OR = 0.69; OR = 0.60, respectively). Associations between family history of breast cancer and OC, regular exercise (≥5/week), and artificial abortion and OC were similar across all subtypes (OR = 3.92; OR = 0.41; OR = 0.72, respectively).

**Conclusion:**

A heterogeneous association between some risk factors and the incidence of each subtype of epithelial OC was observed, suggesting that the carcinogenic mechanisms of each subtype may be partly different.

## Graphical abstract

SCLC patients with TGRmax > −34.3%/m had worse PFS and OS in first‐line immunotherapy plus platin‐based chemotherapy.

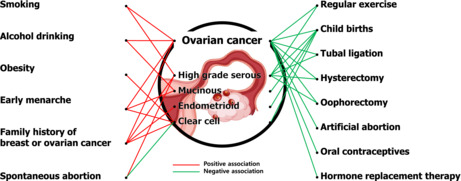

## 1 INTRODUCTION

Globally, ovarian cancer (OC) is one of the most common cancers and the seventh leading cause of cancer‐related deaths in women.^1^ In Korea, 2888 new cases of OC and 1271 deaths caused by OC were estimated in 2019.^2^, ^3^ There are some differences in the incidence of OC based on ethnicity, and Asians, including Koreans, have a lower incidence rate of OC than non‐Hispanic Whites,^4^ despite globalization.^5^ However, despite the lower incidence rate of OC in Asian, the distributions of histologic subtypes are reported to be different according to their race/ethnicity.^4^

Among all types of OC, epithelial OC (EOC) accounts for 90% of the cases.^6^ EOC can be classified into four subtypes according to its origin: serous type, mucinous type (MUC), endometrioid type (END), and clear cell type (CLC).^7^ Serous OC is more specifically categorized as high‐grade serous type (HGS) and low‐grade serous type (LGS). The EOC subtypes are heterogeneous not only in their cellular origin but also in their distribution. HGS may originate from the epithelium of the fallopian tube.^8^ The origin of LGS is associated with stepwise progression from benign through borderline tumor to noninvasive and invasive.^8^ Serous carcinoma accounts for nearly 48% of cases in Korea and most prevalent serous carcinoma is HGS.^9^ END and CLC originate from endometriotic lesions, and each account for nearly 9% of the total EOC incidence in Korea.^9^ However, the origin of MUC, accounting for 16.2% of the total EOC incidence in Korea, has not been well established.^9^ These subtypes are heterogeneous in their clinical course, with the worst clinical course seen in the HGS subtype.^10^ Moreover, the four subtypes have different molecular alterations and gene expression, and different carcinogenic mechanisms across the subtypes have been proposed based on their genetic and molecular backgrounds.^10^ If each subtype has different carcinogenic mechanisms, the major risk factors may also differ for each subtype. Even if exposed to the same risk factors, a stronger risk is observed in certain subtypes that are heterogeneous due to the differences in underlying biological carcinogenic processes; however, in other subtypes, no such association or a reduced risk may be observed.

In regard to the carcinogenic agents for OC, the International Agency for Research on Cancer (IARC) found sufficient evidence that estrogen menopausal therapy causes carcinogenesis in humans.^11^ However, previous epidemiological studies have proposed that several reproductive factors that can affect endogenous or exogenous estrogen levels are complex factors involved in the carcinogenesis of OC.

Most previous studies on the risk factors for OC have been published in Europe and North America, possibly because of the low incidence of OC among Asians. In most Asian countries, including Korea, many studies have focused on the prognostic factors for OC, but few have examined risk factors.^12^, ^13^, ^14^ In a pooled study on the association between OC and various factors, stratified analysis was performed on the Asian/Pacific race; however, no association with OC was observed in terms of risk factors for individuals of Asian/Pacific race, and parity and contraceptive use among Asians were excluded from the analysis.^15^

We hypothesized that there would be some heterogeneity in the association between risk factors and the different subtypes of EOC, based on the recently proposed hypotheses concerning varying mechanisms of carcinogenesis in the different subtypes. Therefore, this study aimed to investigate risk factors for the overall incidence of EOC and its subtypes in the Korean epithelial ovarian cancer study (Ko‐EVE) and to examine the heterogeneity in the risk factors associated with the incidence of EOC based on its subtypes.

## 2 METHODS

### 2.1 Study design and population

**FIGURE 1:**
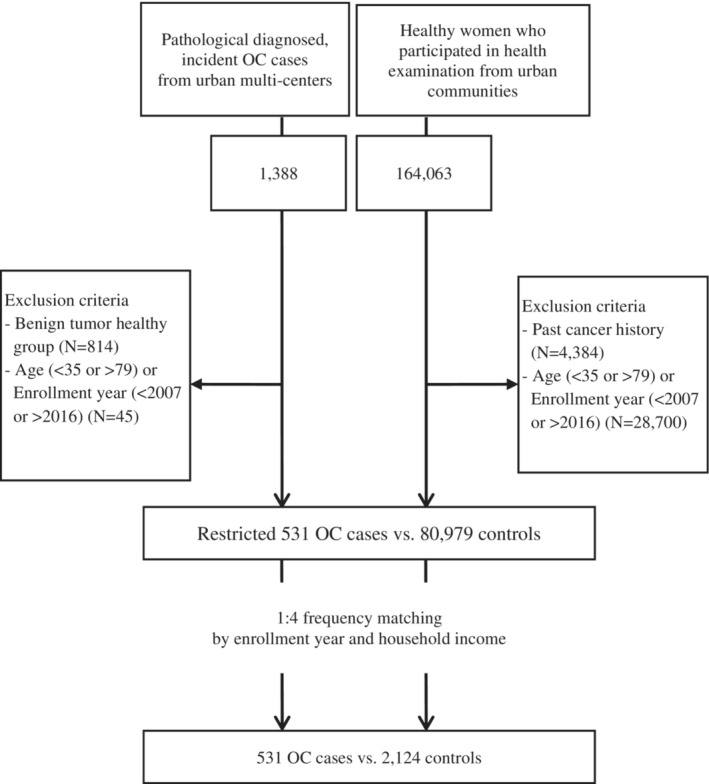
Flow chart of matching.

The Ko‐EVE study has a complex design and comprises prospective cohort and case–control studies based on the data obtained from patients with EOC.^16^ In this database, patients with EOC were enrolled from 2008 to 2015 from seven general hospitals located in Seoul and Gyeonggi‐do, Korea, namely Seoul National University Hospital, Samsung Medical Center, Asan Medical Center, National Cancer Center, Gangnam Severance Hospital, Kyung Hee University Hospital at Gangdong, and Cheil General Hospital. We selected patients with EOC, aged over 20 years, who were histologically confirmed as having stages I–III EOC by pathologists at the time of enrollment.^16^ In Korea, the number of patients with OC has been limited to study the epidemiological research. However, it would be difficult to recruit patients with stage IV cancer considering the consent to biospecimen collection. Therefore, when the researchers designed the study, they did not consider the patients with EOC who were diagnosed with stage IV cancer for enrollment in the study. The histological subtype was confirmed by pathologists. We performed frequency matching for cases and controls with a 1:4 ratio using the enrollment year and household income as variables. The reason for selection of two matching variables was to minimize the confounding effect. When we did preliminary analysis before matching, the significant differences between case and control group were observed for two variables. Finally, we selected 531 patients with EOC and 2124 controls. (Figure 1) This study was approved by Seoul National University College of Medicine/Seoul National University Hospital Institutional Review Board (IRB) (IRB no. 0910‐024‐296).

Healthy controls were enrolled during the same period and comprised women who did not have OC, who underwent screening at general hospitals in Korean cities, including the aforementioned hospitals from which OC patients were enrolled.^16^, ^17^ The control group was derived from a general population‐based cohort, which did a general health screening including blood test and urinalysis. In this study, self‐reported questionnaire data was only used for the control group. The criterion for selecting control group was the being from the same region as the case group and availability to obtain information about the necessary variables for this study. Details on the study design and methodologies of the Ko‐EVE study have been described previously.^16^

### 2.2 Data collection

Information from participants were collected using questionnaires, anthropometric measurements, physical examinations, laboratory tests, tumor marker tests, urine tests, electrocardiogram (ECG) results, bone densitometer (BMD) results, and pelvic and vaginal ultrasound examinations. The questionnaires included demographic information, history of hospitalization, lifestyle factors, family history of cancer, medical history, medication history, and reproductive history. In addition, in the baseline case report form (CRF), results of laboratory tests, urine test, chest X‐ray, ECG, BMD, gynecologic symptoms, description of the radiologic diagnosis, and surgical and pathological reports were collected. More information on the Ko‐EVE study has been described elsewhere.^16^

### 2.3 Statistical analysis

The baseline differences were compared between cases and controls using Pearson's Chi‐squared test for categorical variables and Student's *t*‐test for continuous variables. A conditional logistic regression model was used to calculate the odds ratios (ORs) and 95% confidence intervals (CIs) to evaluate the association between risk factors and the overall risk of EOC. An unconditional logistic regression model was used to evaluate the association between risk factors and the incidence of EOC by subtypes because the matching of the case–control design was broken. We selected the following factors as potential confounders in the multiple logistic regression model: (1) age and education as covariates related to affecting most reproductive and lifestyle factors; (2) oral contraceptive (OCP) use because estrogen‐based contraception was proposed as a carcinogen for OC by the IARC^11^; (3) family history of breast cancer and OC, because family history is known to be a highly significant factor affecting OC incidence, along with body mass index (BMI)^18^, ^19^, ^20^, ^21^, ^22^, ^23^, ^24^; (4) obesity as it was one of the suggested risk factors for OC in previous studies^6^; (5) cigarette smoking as one of the carcinogenic agents with sufficient evidence in humans according to IARC^11^; (6) and alcohol drinking as a controversial factor for OC incidence.^25^ If the number of observed cases was less than five, we performed the Cochran–Mantel–Haenszel test to calculate the ORs and 95% CIs. All statistical analyses were performed using SAS software (version 9.4; SAS Institute). For adjustment for multiple comparisons, we have corrected for multiple comparisons by using an adjusted alpha of 0.0167 (0.05/3 tests for HGS‐mucinous, HGS‐endometrioid, and HGS‐clear cell).

## 3 RESULTS

Between the case and control groups, there was a statistically significant difference in age, duration of OCP use, and history of hypertension (*p* < 0.05). (Table 1) Cigarette smoking was significantly associated with an increased risk of overall EOC incidence (OR = 1.78, 95% CI = 1.03–3.07), especially the incidence of HGS OC (OR = 2.69, 95% CI = 1.15–6.30). Higher pack‐years of smoking (≥10 pack‐years) had a much stronger association with the risk elevation of EOC and HGS (OR = 3.14, 95% CI = 1.32–7.44; OR = 9.59, 95% CI = 2.82–32.63, respectively). Alcohol consumption was associated with an increased risk of overall EOC and MUC incidence (OR = 1.31, 95% CI = 1.02–1.67; OR = 3.63, 95% CI = 1.39–9.49, respectively). The risk of MUC was strongly associated with alcohol consumption, regardless of the dose consumed per week (<5 times per week: OR = 4.95; ≥5 times per week: OR = 4.29). Obesity (BMI ≥30 kg/m^2^) was positively associated with risk of CLC (OR = 4.57, 95% CI = 1.06–19.77), and the risk association of CLC showed heterogeneity from the risk of HGS (*p* = 0.045). Regular, hyper sweating exercise performed ≥5 times per week was negatively associated with the risk of EOC (OR = 0.41, 95% CI = 0.28–0.60) (Table 2), and longer duration of exercise (>1.5 h) per exercise session was also negatively associated with the risk for EOC (OR = 0.60, 95% CI = 0.41–0.86). Similar associations were observed between HGS and other subtypes. Family history of breast cancer with or without OC had strongly positive association with the risk of EOC (OR = 6.50, 95% CI = 4.38–9.65), especially among patients with HGS (OR = 9.14, 95% CI = 5.05–16.55). Tubal ligation and hysterectomy prior to EOC diagnosis was associated with a reduced risk of EOC and HGS (EOC overall: OR = 0.17, 95% CI = 0.10–0.31 and OR = 0.34, 95% CI = 0.19–0.62; HGS: OR = 0.14, 95% CI = 0.05–0.39 and OR = 0.23, 95% CI = 0.07–0.73). Oophorectomy was associated with a significantly reduced risk of HGS (OR = 0.28, 95% CI = 0.08–0.97). The association between developing HGS and tubal ligation was heterogeneous from the risk of developing CLC (*p* < 0.01); the risks of CLC based on the factor showed an increased association pattern (OR = 1.58). (Table 2) However, in the case of hysterectomy and oophorectomy, with the consideration of multiple comparison, there were no heterogeneity between HGS and CLC (*p* = 0.049 and *p* = 0.05, respectively).

**TABLE 1:** Selected characteristics of ovarian cancer cases and controls in the Korean epithelial ovarian cancer study (Ko‐EVE), 2008–2015.

	OC cases (*N* = 531)	Controls (*N* = 2124)
	Mean (SD)	Mean (SD)
Age (years)^a^	52.4 (10.3)	51.7 (7.7)
Height (cm)	156.7 (5.8)	157.2 (5.4)
Weight (kg)	57.0 (9.1)	57.6 (8.0)
Body mass index (kg/m^2^)	23.2 (3.6)	23.3 (3.0)
Age at menarche (years)	15.1 (2.4)	15.0 (1.8)
Age at menopause (years)^b^	49.1 (7.0)	49.4 (4.8)
	Median (IQR)	Median (IQR)
Breastfeeding duration (months)^c^	13.0 (2.0–25.0)	13.0 (3.0–24.0)
Duration of oral contraceptive use (months)^a^	5.0 (1.0–12.0)	9.0 (2.0–12.0)
Duration of HRT (months)^c^	12.5 (3.0–48.0)	18.0 (4.5–60.0)
	*N* (%)	*N* (%)
Household income^d^		
<2000$	111 (20.9)	444 (20.9)
2000–3999	187 (35.2)	748 (35.2)
≥4000	233 (43.9)	932 (43.9)
Education ≥9 years	296 (73.3)	1586 (74.9)
Ever‐smokers	20 (5.0)	67 (3.2)
Ever‐drinkers	156 (38.2)	714 (33.6)
Regular exercise enough to sweat	194 (47.1)	1088 (51.2)
Hypertension^a^	107 (21.2)	329 (15.5)
Diabetes mellitus	28 (5.5)	104 (4.9)
Benign thyroid diseases	28 (5.5)	111 (5.2)

Abbreviations: HRT, postmenopausal hormone replacement therapy; IQR, interquartile range; OC, ovarian cancer; SD, standard deviation.

^a^ *p*‐value < 0.05 in the difference of distribution between cases and controls by Student's *t*‐test for continuous variables or chi‐square test for categorical variables.

^b^ Among postmenopausal women.

^c^ Among parous women.

^d^ Matched variable.

**TABLE 2:** Lifestyle factors, family histories, and surgical histories on the risk of overall epithelial ovarian cancer (EOC) and EOC subtypes in the Korean epithelial ovarian cancer study (Ko‐EVE), 2008–2015.

	Case	Control	EOC	HGS	MUC	END	CLC
			OR (95% CI)^a^	OR (95% CI)^a^	OR (95% CI)^a^	OR (95% CI)^a^	OR (95% CI)^a^
Cigarette smoking							
Never	382	2052	1.00	1.00	1.00	1.00	1.00
Ever	20	60	1.78 (1.03–3.07)	2.69 (1.15–6.30)	2.83 (0.45–17.66)	4.24 (0.73–24.46)	3.47 (0.31–44.24)
Smoking pack‐year							
Never	382	2052	1.00	1.00	1.00	1.00	1.00
<10 pack‐years	8	49	1.00 (0.45–2.19)	0.74 (0.16–3.39)	‐	2.53 (0.39–16.34)	3.22 (0.29–36.17)
≥10	9	18	3.14 (1.32–7.44)	9.59 (2.82–32.63)	1.81 (0.03–96.82)	8.01 (2.71–23.72)	5.74 (0.45–73.16)
Alcohol drinking							
Never	252	1409	1.00	1.00	1.00	1.00	1.00
Ever	156	714	1.31 (1.02–1.67)	1.37 (0.95–2.17)	3.63 (1.39–9.49)	0.75 (0.29–1.95)	1.77 (0.71–4.40)
Alcohol dose							
Never	252	1409	1.00	1.00	1.00	1.00	1.00
<5 g/week	79	432	1.12 (0.82–1.51)	1.48 (0.89–2.45)	4.50 (1.59–12.74)	0.42 (0.09–1.94)	2.45 (0.77–7.81)
≥5	49	281	1.13 (0.66–1.95)	0.46 (0.13–1.67)	4.29 (0.98–22.19)	0.78 (0.10–6.09)	1.38 (0.16–11.57)
Body mass index							
<18.5 kg/m^2^	32	63	1.80 (0.94–3.43)	1.47 (0.60–3.63)	1.66 (0.20–14.20)	0.81 (0.09–7.01)	0.13 (0.01–5.75)
18.5–22.9	246	1009	1.00	1.00	1.00	1.00	1.00
23–24.9	111	504	0.94 (0.70–1.28)	0.96 (0.59–1.56)	1.06 (0.30–3.78)	0.81 (0.25–2.64)	0.51 (0.14–1.92)
25–29.9	125	485	1.20 (0.90–1.60)	0.89 (0.55–1.44)	1.85 (0.65–5.25)	1.09 (0.38–3.11)	0.69 (0.22–2.19)
≥30	16	59	0.98 (0.48–2.02)	0.70 (0.21–2.40)^b^	4.95 (0.48–50.57)	1.68 (0.21–13.65)	4.57 (1.06–19.77)^b^
Regular exercise							
0–2 times/week	294	1265	1.00	1.00	1.00	1.00
3–4	71	413	0.78 (0.58–1.06)	0.71 (0.43–1.18)	0.35 (0.08–1.56)	1.06 (0.29–3.91)	1.96 (0.74–5.21)
≥5	40	420	0.41 (0.28–0.60)	0.36 (0.19–0.70)	0.39 (0.05–2.85)	0.17 (0.02–1.32)	0.76 (0.16–3.58)
Family history of BC and/or OC							
No	339	2067	1.00	1.00	1.00	1.00	1.00
Yes	61	57	6.50 (4.38–9.65)	9.14 (5.05–16.55)	1.33 (0.01–25.80)	7.17 (1.80–28.49)	5.53 (0.94–32.79)
Family history of BC							
No	353	2070	1.00	1.00	1.00	1.00	1.00
Yes	43	54	3.92 (2.47–6.21)	7.01 (3.65–13.47)	1.33 (0.01–29.87)	5.19 (1.31–20.64)	5.53 (0.94–32.79)
Tubal ligation							
Never	400	1681	1.00	1.00	1.00	1.00	1.00
Ever	15	439	0.17 (0.10–0.31)	0.14 (0.05–0.39)^b^	0.26 (0.03–2.19)	0.35 (0.04–2.84)	1.58 (0.53–4.71)^b^
Hysterectomy							
Never	463	1912	1.00	1.00	1.00	1.00	1.00
Ever	20	209	0.34 (0.19–0.62)	0.23 (0.07–0.73)^b^	0.34 (0.03–3.30)	1.74 (0.49–6.17)	1.63 (0.38–7.02)^b^
Oophorectomy							
Never	484	1992	1.00	1.00	1.00	1.00	1.00
Ever	22	129	0.72 (0.42–1.23)	0.28 (0.08–0.97)^b^	0.74 (0.09–5.84)	0.78 (0.10–6.11)	1.82 (0.43–7.68)^b^

Abbreviations: BC, breast cancer; CI, confidence interval; CLC, clear cell; END, endometrioid; HGS, high grade serous; MUC, mucinous; OC, ovarian cancer; OR, odds ratio.

^a^ For total OC, conditional logistic regression model stratified by enrollment year and household income and adjusted for age, educational levels, oral contraceptive use, family history of breast cancer and ovarian cancer, and body mass index was performed. For OC subtype, unconditional logistic regression model adjusted for age, educational levels, oral contraceptive use, family history of breast cancer and ovarian cancer, and body mass index was performed.

^b^ *p*‐heterogeneity between two ORs in the specific subtype and HGS type: BMI ≥ 30, *p* = 0.045 in CLC; Tubal ligation, *p* < 0.01 in CLC; Hysterectomy, *p* = 0.049 in CLC; Oophorectomy, *p* = 0.05 in CLC.

Earlier age at menarche (≤13 years) was associated with a 1.48‐fold higher risk of the overall incidence of EOC (OR = 1.48, 95% CI = 1.05–2.09), as compared to later age at menarche (≥16 years). Early menarche was generally associated with an increased risk for each EOC subtype; in particular, a highly positive association, of at least threefold or higher, was observed with the incidence of CLC and MUC (OR = 6.11, 95% CI = 1.53–24.42; OR = 3.23, 95% CI = 0.98–10.86, respectively; heterogeneity with HGS, *p* = 0.07 for MUC and *p* = 0.07 for CLC). (Table 3) Parity was negatively associated with the risk of EOC by 0.24‐fold (OR = 0.24, 95% CI = 0.16–0.37), regardless of the subtype. Undergoing childbirth two or more than two times was negatively associated with the risk of EOC (one childbirth: OR = 0.33, 95% CI = 0.20–0.55; two or more childbirths: OR = 0.23, 95% CI = 0.15–0.35). In particular, two or more childbirths had a stronger negative association with the risk of CLC and END subtypes (OR = 0.11, 95% CI = 0.04–0.35; OR = 0.12, 95% CI = 0.02–0.37, respectively; heterogeneity with HGS, *p* = 0.05 for CLC and *p* = 0.044 for END). Practicing breastfeeding and its duration was not significantly associated with the risk of EOC, regardless of the EOC subtype. Spontaneous abortion showed negative association only in CLC (OR = 0.34, 95% CI = 0.13–0.90); the risk in CLC was different from that in the HGS subtype (OR = 1.43, 95% CI = 0.96–2.15, *p* = 0.01). Artificial abortion was negatively associated with a risk of overall EOC incidence (OR = 0.72, 95% CI = 0.54–0.97), with a non‐significant but reduced risk observed for all EOC subtypes. The use of OCP and hormonal replacement therapy (HRT) was negatively associated with a risk of EOC (OCP: OR = 0.61, 95% CI = 0.40–0.93; HRT: OR = 0.51, 95% CI = 0.32–0.80). The association between OCP and HRT was similar in HGS (OR = 0.69, OR = 0.60, respectively); however, a non‐significant but high risk was observed in the incidence of CLC.

**TABLE 3:** Reproductive factors on the risk of overall epithelial ovarian cancer (EOC) and EOC subtypes in the Korean epithelial ovarian cancer study (Ko‐EVE), 2008–2015.

	Case	Control	EOC	HGS	MUC	END	CLC
			OR (95% CI)^a^	OR (95% CI)^a^	OR (95% CI)^a^	OR (95% CI)^a^	OR (95% CI)^a^
Age at menarche (years)							
≤13 years	108	412	1.48 (1.05–2.09)	1.37 (0.85–3.10)^d^	3.23 (0.98–10.86)^d^	1.14 (0.36–3.07)	6.11 (1.53–24.42)^d^
14–15	198	985	0.98 (0.74–1.32)	0.90 (0.57–1.41)	0.99 (0.29–3.35)	0.25 (0.07–0.83)	1.35 (0.32–5.62)
≥16	169	712	1.00	1.00	1.00	1.00	1.00
Age at menopause^b^							
<49 years	87	373	1.00	1.00	1.00	1.00	1.00
49–51	96	381	0.96 (0.66–1.40)	1.15 (0.65–2.03)	0.32 (0.06–1.66)	0.66 (0.18–2.40)	0.75 (0.16–3.56)
≥52	108	388	0.91 (0.62–1.34)	0.66 (0.36–1.21)	0.35 (0.09–1.37)	0.40 (0.09–1.78)	0.70 (0.13–3.80)
Number of childbirths							
Nullipara	56	68	1.00	1.00	1.00	1.00	1.00
1	65	244	0.33 (0.20–0.55)	0.43 (0.18–1.00)	0.42 (0.10–1.86)	0.18 (0.04–0.80)	0.34 (0.08–1.40)
2+	381	1771	0.23 (0.15–0.35)	0.25 (0.19–0.50)^d^	0.14 (0.03–0.52)	0.11 (0.04–0.35)^d^	0.12 (0.02–0.37)^d^
First childbirth age^c^							
<24 years	95	401	1.00	1.00	1.00	1.00	1.00
24–29	221	1326	0.76 (0.53–1.10)	0.64 (0.37–1.11)	1.08 (0.22–5.26)	5.28 (0.58–47.68)	2.69 (0.31–23.66)
≥30	65	282	0.99 (0.64–1.53)	0.87 (0.45–1.69)	4.38 (0.91–21.10)	4.29 (0.38–48.25)	3.66 (0.38–35.34)
Last childbirth age^c^							
<29 years	140	658	1.00	1.00	1.00	1.00	1.00
29–34	205	985	1.01 (0.72–1.41)	0.86 (0.51–1.46)	1.16 (0.32–4.41)	1.39 (0.43–4.54)	1.35 (0.36–5.17)
≥35	52	124	1.68 (1.07–2.64)	1.25 (0.60–2.60)	1.01 (0.11–9.07)	1.00 (0.10–12.80)	2.28 (0.46–11.37)
Breastfeeding^c^							
Never	71	349	1.00	1.00	1.00	1.00	1.00
Ever	404	1707	0.83 (0.60–1.14)	0.90 (0.53–1.54)	0.49 (0.15–1.59)	0.73 (0.23–2.37)	0.44 (0.15–1.26)
Breastfeeding duration^c^							
Never	71	349	1.00	1.00	1.00	1.00	1.00
<6 months	66	339	0.84 (0.55–1.27)	1.32 (0.68–2.56)	0.23 (0.02–2.26)	1.34 (0.34–5.37)	0.37 (0.07–1.94)
≥6	338	1368	0.83 (0.59–1.15)	0.80 (0.46–1.39)	0.55 (0.17–1.82)	0.57 (0.16–1.97)	0.46 (0.15–1.38)
Spontaneous abortion							
Never	209	849	1.00	1.00	1.00	1.00	1.00
Ever	291	1273	1.02 (0.80–1.29)	1.43 (0.96–2.15)^d^	0.48 (0.19–1.19)	0.87 (0.36–2.08)	0.34 (0.13–0.90)^d^
Artificial abortion							
Never	414	1666	1.00	1.00	1.00	1.00	1.00
Ever	81	16,555	0.72 (0.54–0.97)	0.64 (0.34–1.03)	0.61 (0.16–2.31)	0.63 (0.18–2.16)	0.22 (0.03–1.64)
OCP use							
Never	450	1862	1.00	1.00	1.00	1.00	1.00
Ever	49	258	0.61 (0.40–0.93)	0.69 (0.34–1.40)	1.03 (0.23–4.58)	0.49 (0.09–2.64)	1.86 (0.52–6.67)
OCP duration							
Never	450	1862	1.00	1.00	1.00	1.00	1.00
<10 months	21	122	0.59 (0.33–1.06)	0.63 (0.23–1.76)	1.13 (0.14–9.25)	0.49 (0.05–5.04)	2.26 (0.48–10.56)
≥10	17	121	0.50 (0.26–0.96)	0.50 (0.16–1.54)	2.01 (0.44–9.14)	0.48 (0.05–4.96)	1.40 (0.18–11.00)
HRT use^b^							
Never	228	894	1.00	1.00	1.00	1.00	1.00
Ever	36	264	0.51 (0.32–0.80)	0.60 (0.32–1.12)	0.46 (0.09–2.27)	0.61 (0.13–2.90)	1.15 (0.23–5.85)
HRT duration^b^							
Never	228	894	1.00	1.00	1.00	1.00	1.00
≤12 months	17	120	0.56 (0.30–1.04)	0.63 (0.26–1.51)	0.43 (0.05–3.95)	0.70 (0.09–5.69)	1.33 (0.16–11.35)
>12	18	136	0.44 (0.23–0.84)	0.55 (0.24–1.27)	0.49 (0.06–3.94)	0.56 (0.07–4.53)	1.02 (0.12–8.90)

Abbreviations: CI, confidence interval; CLC, clear cell; END, endometrioid; HGS, high grade serous; HRT, hormone replacement therapy; MUC, mucinous; OCP, oral contraceptive; OR, odds ratio.

^a^ For total OC, conditional logistic regression model stratified by enrollment year and household income and adjusted for age, educational levels, oral contraceptive use, family history of breast cancer and ovarian cancer, and body mass index was performed. For OC subtype, unconditional logistic regression model adjusted for age, educational levels, oral contraceptive use, family history of breast cancer and ovarian cancer, and body mass index was performed.

^b^ Among postmenopausal women.

^c^ Among parous women.

^d^ *p*‐heterogeneity between two ORs in the specific subtype and HGS type: for age at menarche, *p* = 0.05 in MUC, *p* = 0.07 in CLC; for number of childbirths, *p* = 0.044 in END, *p* = 0.05 in CLC; for spontaneous abortion, *p* = 0.01 in CLC.

## 4 DISCUSSION

In our study, according to the OC subtypes, alcohol consumption and obesity were positively associated with the incidence risk of specific EOC types (alcohol consumption for MUC incidence and obesity for CLC incidence). Some risk factors for EOC were associated with either an increased or decreased risk, regardless of the subtype; however, stronger associations with specific subtypes were also observed (cigarette smoking and family history of breast cancer and OC, increased the incidence of HGS; early menarche increased the incidence of CLC and MUC; and parity (≥2 childbirths) decreased the risk of END and CLC). Other risk factors were observed to have similar associations with overall incidence of EOC and HGS; however, heterogeneity between the incidence risk of CLC and HGS was also observed due to differences in associations. For instance, tubal ligation, showed a negative association with HGS and EOC, whereas a non‐significant positive association with CLC was observed. Parity, regular and hyper sweating exercise, and artificial abortion were preventable factors for EOC incidence, regardless of the subtype.

Weak heterogeneity patterns were observed for each subgroup of EOC, and the specific patterns were as follows: (1) Earlier age at menarche was much stronger in CLC and MUC; (2) A reduction in the incidence risk of EOC after experiencing two or more childbirths was much stronger in the END and CLC subtypes; (3) Spontaneous abortion showed a significantly reduced risk of CLC incidence, but a higher risk of HGS incidence; (4) Exogenous hormonal use (such as OCP and HRT), surgery concerning the reproductive organs prior to an EOC diagnosis, and regular exercise were associated with risk reduction in most subtypes, but opposingly showed an elevated risk of developing CLC, albeit non‐significantly; (5) Alcohol consumption was associated with a higher risk of MUC; and (6) Obesity was associated with a higher risk of developing CLC.

As shown in a meta‐analysis on OC, older age at menarche by 1‐year was associated with a 0.97‐fold decreased risk of developing EOC.^12^ Parity and breastfeeding have also been reported to be protective factors.^13^ Although parity has a weaker effect on the negative association of HGS, it has a stronger negative association in END and CLC subtypes (50%–70% reduction).^26^ According to histological subtypes, early age at menarche was strongly associated with a risk reduction in CLC incidence,^26^ which is consistent with our results. However, no reports have shown an association between MUC and early age at menarche.

Parity has been reported to be a protective factor for developing all subtypes of EOC. Considering the histological subtypes, parity showed a weaker effect on the risk of developing serous OC; however, a stronger effect against the risk of developing END and CLC subtypes has been reported.^26^ Similar results were observed in the present study. Previous studies have shown that breastfeeding is a significant protective factor for OC incidence^6^, ^18^, ^26^; however, in this study, breastfeeding had a non‐significant effect on the risk reduction of EOC incidence, regardless of the EOC subtype. The impact of spontaneous or artificial abortion has not been clearly validated.^6^ In this study, a slightly significant increased risk was observed in HGS carcinoma. On the other hand, a decreased risk was observed in CLC incidence after spontaneous abortion. In addition, considering the overall EOC incidence, artificial abortion was observed to be a significant protective factor.

Previous studies have shown that OCPs, which regulate the ovulation cycle, are associated with a reduced risk of OC.^26^ However, previously reported associations between postmenopausal HRT and the risk of EOC incidence remain controversial. This may be because previous studies that mostly used unopposed estrogen therapy, that is, combined estrogen and progesterone therapy, showed a weak association between the reduced risk of EOC incidence and HRT, while a few recent studies that used various components of HRT presented a strong association with increased risk of EOC incidence.^6^ In our findings, both OCP and HRT reduced the risk of EOC. A significant negative association in the risk of EOC incidence was observed for HRT, especially in individuals who used HRT for over 12 months; however, there was a non‐significant association with the risk of each subtype. This result can be discussed at two points. First, the overall HRT use in Korea and OC risk may be negatively associated and there may be a dose‐response effect. Second, individuals who continuously receive HRT after menopause might actively visit the hospital and be healthy. Therefore, it may have been shown that the longer treatment had negative association with OC. OCP use was also a protective factor for overall EOC incidence, especially in individuals who used OCPs for 10 months or longer. The results for HRT may reflect the prescription patterns of HRT in Korea. In Korea, most of the prescribed HRTs are a combination of estrogen and progesterone (53%) and tibolone and estrogen (40%), which shows progestogenic and androgenic properties in metabolism, and only 7% of individuals are prescribed only estrogen in Korea.^27^ In addition, OCP use was inversely associated with the incidence of HGS and CLC subtypes^26^ in a consortium study, which was similar to our findings. In a previous study, an increased risk of serous and END carcinomas was observed in those who used HRT. In the case of tubal ligation and hysterectomy, protective effects were observed for the overall incidence of EOC along with HGS incidence. Oophorectomy was observed to have a protective effect against HGS incidence. On the other hand, according to previous studies, tubal ligation was shown to offer 25%–50% negative association for all invasive OCs, null‐weak protection for HGS and MUC, and 50% or stronger negative association for END and CLC subtypes of OC.^6^ For a long time, family history has been considered as one of the most important risk factors for OC.^6^, ^28^ In a previous study, an increased risk was observed in those with a family history of breast cancer, for all invasive, serous, and END carcinomas, with statistically significant *p*‐heterogeneity.^26^ Likewise, in this study, a family history of breast cancer and OC was observed to have a strong effect on the risk of developing overall EOC, HGS, and END carcinomas.

According to other cohort study which included 300,398 Norwegian women and consortium study which included 1.3 million women from 21 studies, there was a positive association between smoking and MUC.^29^ In other subtypes, there was no association. However, in this study, a positive association was observed between smoking and HGS, not MUC. This might be due to the fact that the distribution of subtypes is different between Korean and western population or the fact that data of this study is limited to evaluate smoking. Further studies to verify the association between cigarette smoking and each histologic subtype of OC with consideration of race/ethnicity are needed. In previous studies, alcohol consumption has not shown consistent associations.^6^, ^30^, ^31^, ^32^ In this study, alcohol consumption showed an increased risk for developing overall EOC and MUC. Even though exercise might reduce adipose tissue, estrogen levels, and chronic inflammation^33^, ^34^ results of previous studies on physical activity and exercise have not been consistent.^6^ According to a previous consortium study that included 8309 patients and 12,612 controls, physical inactivity was observed to have a significant risk for the overall incidence of EOC, along with the incidence of HGS, MUC, END, and CLC subtypes.^35^ However, there is a lack of sufficient studies that could verify the benefits of physical activity on the risk reduction of OC.^6^, ^36^, ^37^

According to this study, body mass index (BMI) <18.5 and ≥30 kg/m^2^ was shown to have a significantly higher risk of the overall incidence of EOC and CLC, respectively. On the other hand, a previous meta‐analysis showed that higher BMI levels are associated with OC risk.^38^ In a previous review, BMI had a weak risk of 25% or less for all OC subtypes, except for HGS.^6^

Our study has several limitations. One of which is information bias, including recall and memory decay bias, because most variables used in this study were obtained from the self‐reported questionnaire surveys. There was no consideration of stage IV patients because only stage I–III patients were included in the case group. Various demographics variables such as comorbidities and use of medication could not be considered because of limitation of derived data. Further, considering the adjustment for multiple comparison to minimize the type I error, the type II error might have been increased.^39^ Although most HRTs are a combination of estrogen and progesterone or tibolone, only 7% of individuals use estrogen alone in Korea.^27^ However, in our data, most participants could not distinguish the detailed HRT components; therefore, we could not avoid the bias for HRT. Due to a small number of patients with LGS carcinoma, we could not include LGS in the analysis. However, we can consider HGS as a serous carcinoma because most serous carcinomas are HGS. Since the number of women with factors such as cigarette smoking or family history of OC was small, their 95% CIs were calculated for a wide range of MUC, END, and CLC subtypes, which limited the evaluation of heterogeneity across EOC subtypes. Nevertheless, our study also has several strengths. To the best of our knowledge, this study is the first to present a wide range of risk factors for OC in Korea, including reproductive and hormonal factors, lifestyle factors, and anthropometric variables, along with stratifying these risk factors based on EOC subtypes, even though the number of cases belonging to each EOC subtype was small. Although this study included a small number of OC patients because the patients enrolled were Korean, it contains a relatively higher number of patients with the CLC subtypes than that in studies from Western countries.^4^ In a previous study, it was reported that the percentage of patients with CLC among all patients with OC was higher among Asians (approximately 11%) than that in Western countries (3%–5%).^40^, ^41^

Most previous epidemiological studies on overall OC or EOC have not evaluated the association between risk factors and each subtype of OC or EOC.^6^ In those studies, the association between risk factors for OC, a single disease entity, has been analyzed, whereas the present study was focused on OC having different carcinogenic mechanisms depending on its histological type. Very few studies have evaluated the risk factors for overall EOC incidence and EOC incidence based on its histologic subtype in Asia. In this study, we confirmed that there are risk factors that similarly affect EOC incidence, regardless of the histological subtype; however, that there are risk factors that have distinct effects in different histological subtypes of EOC. Considering that it is anatomically difficult to detect OC earlier, in the preclinical phase, it is usually detected at a late stage, leading to a poor prognosis. Therefore, primary modes of prevention are crucial than secondary or tertiary prevention methods.^42^

This study demonstrates a heterogeneous association between the risk of developing OC based on each subtype of EOC and certain risk factors, suggesting that risk factors for each subtype may act heterogeneously or may have partially different mechanisms of carcinogenesis.

## AUTHOR CONTRIBUTIONS

**Soseul Sung:** Conceptualization (equal); data curation (equal); formal analysis (lead); investigation (lead); methodology (lead); writing – original draft (lead); writing – review and editing (equal). **Youjin Hong:** Writing – review and editing (equal). **Byoung Gie Kim:** Data curation (equal); funding acquisition (equal); project administration (equal); writing – review and editing (equal). **Ji‐Yeob Choi:** Conceptualization (equal); project administration (equal); writing – review and editing (equal). **Jae‐Weon Kim:** Data curation (equal); project administration (equal); writing – review and editing (equal). **Sang‐Yoon Park:** Project administration (equal); writing – review and editing (equal). **Jae Hoon Kim:** Data curation (equal); project administration (equal); writing – review and editing (equal). **Yong Man Kim:** Data curation (equal); project administration (equal); writing – review and editing (equal). **Jong‐Min Lee:** Data curation (equal); project administration (equal); writing – review and editing (equal). **Tae Jin Kim:** Data curation (equal); project administration (equal); writing – review and editing (equal). **Sue K. Park:** Conceptualization (equal); data curation (equal); formal analysis (supporting); funding acquisition (equal); investigation (supporting); methodology (supporting); project administration (equal); writing – review and editing (equal).

## CONFLICT OF INTEREST

The authors declare no potential conflict of interest.

## ETHICS STATEMENT

This study was approved by Seoul National University College of Medicine/Seoul National University Hospital Institutional Review Board (IRB) (IRB no. 0910–024‐296).

## FUNDING INFORMATION

This study (Ko‐EVE) was supported by a grant from the Korea Health Technology R&D Project through the Korea Health Industry Development Institute (KHIDI), and the National R&D Program for Cancer Control, Ministry of Health & Welfare, Republic of Korea (HI16C1127; 0920010). This study was funded by the Korean Foundation for Cancer Research (Grant Number. CB‐2017‐A‐2).

## Data Availability

I confirm that my article contains a Data Availability Statement even if no data is available (list of sample statements) unless my article type does not require one.
